# Comparison of anticoagulation efficacy and safety of different heparin dosages in cardiogenic shock patients supported by intra-aortic balloon pump: A retrospective cohort study

**DOI:** 10.1097/MD.0000000000051386

**Published:** 2026-11-06

**Authors:** Yunkai Li, Weixia Yang, Yiwei Yu, Bingbing Fan, Huijun Dong, Yizhe Wang, Hanxiao Nie, Zhengtao Hao, Zhenzhen Wang, Xingguo Niu, Dalong Zhang

**Affiliations:** a Henan University of Chinese Medicine Fifth Clinical Medical College, Zhengzhou, China.

**Keywords:** anticoagulation management, bleeding, cardiogenic shock, heparin flush, intra-aortic balloon pump, thrombocytopenia, transfusion

## Abstract

The study aimed to evaluate whether a half-dose heparin flush strategy during intra-aortic balloon pump (IABP) support improves safety compared with the standard-dose strategy in patients with cardiogenic shock (CS). In this single-center retrospective cohort study, 128 CS patients who received IABP support between January 2023 and December 2024 were included. Sixty-eight patients received a standard-dose heparin flush, and 60 received a half-dose flush. The primary endpoints were clinically relevant bleeding (Bleeding Academic Research Consortium 2, 3, or 5) and thrombocytopenia (platelet count < 100 × 10^9^/L). Multivariable logistic regression was conducted, adjusting for dual antiplatelet therapy and renal dysfunction. The incidence of clinically significant bleeding events and thrombocytopenia was significantly lower in the half-dose group than in the standard-dose group (*P* < .05). Compared with the standard-dose group, the half-dose heparin flush strategy was significantly associated with a lower risk of clinically significant bleeding (odds ratio = 0.15; 95% confidence interval = 0.03–0.68). Among the secondary outcomes, the half-dose group had a significantly lower transfusion requirement than the standard-dose group (*P* < .001). No significant differences were observed between the 2 groups in activated partial thromboplastin time, activated clotting time, in-hospital mortality, or catheter-related occlusion events. In patients with CS receiving IABP support, the half-dose heparin flush strategy was associated with lower incidences of thrombocytopenia, clinical bleeding, and transfusion requirements. No device-related thrombotic events were observed in either group, suggesting that the half-dose heparin flush strategy may contribute to optimizing anticoagulation management during the peri-support period.

## 1. Introduction

Cardiogenic shock (CS) is a highly lethal clinical syndrome in acute cardiovascular diseases, characterized by insufficient tissue perfusion, organ failure, and progressive circulatory failure.^[1,2]^ Despite significant advancements in percutaneous interventional therapy and drug support techniques in recent years, for patients with extremely unstable hemodynamics, drug therapy alone often fails to rapidly reverse the shock process. The intra-aortic balloon pump (IABP) is one of the most widely used mechanical circulatory support devices in clinical practice and is commonly used for short-term circulatory support in CS patients.^[3]^ Although randomized controlled studies have failed to confirm that IABP can significantly reduce mortality,^[4,5]^ it is still frequently applied in real-world clinical practice,^[5]^ especially in intensive care settings or as a transitional measure for other circulatory support strategies.^[6]^ Therefore, the identification and management of IABP-related complications remain important clinical issues in the treatment of CS.

During IABP support, long-term effective operation highly depends on the patency of the catheter system. To prevent catheter-related thrombosis, heparinized saline is routinely used for continuous catheter flushing to maintain a local anticoagulant microenvironment at the catheter tip.^[7]^ However, there is still no unified standard for the optimal anticoagulation strategy during IABP support, and real-world practice varies substantially across centers and care units.^[8]^ Currently, the selection of heparin concentration in the flushing solution is mostly based on experience, and there is a lack of quantitative research on the impact of different doses on coagulation, bleeding events, and the incidence of systemic platelet count decline.

Platelet count decline and bleeding complications are common problems in CS patients during intensive care unit (ICU) treatment, especially when receiving mechanical circulatory support.^[9]^ The mechanism of thrombocytopenia is complex and may be related to mechanical platelet consumption associated with the device, inflammatory response, infection, and anticoagulation therapy, among other factors.^[10]^ Although heparin-induced immune thrombocytopenia is a serious complication that requires high vigilance,^[11]^ in most CS patients, the more common cause of platelet decline is nonimmune mechanisms. Whether the low-dose heparin exposure from continuous flushing has additional effects on platelet count changes and bleeding risk remains poorly studied.

Although previous studies have explored the relationship between systemic anticoagulation intensity and thrombotic and bleeding events, direct comparative evidence regarding the clinical impact of different heparin doses in IABP flushing solutions, especially their relationship with platelet count decline and bleeding events, is still limited. Therefore, it is necessary to evaluate the safety and efficacy of different heparin flushing strategies in real-world CS populations.

This study aims to retrospectively analyze the application effects of standard-dose 12,500 U/500 mL and half-dose 6250 U/500 mL heparin flushing regimens in CS patients, focusing on evaluating the 2 strategies in maintaining catheter patency (anticoagulation effectiveness) and safety indicators such as platelet count changes, bleeding events, and blood transfusion requirements, with the aim of providing more definite clinical evidence for anticoagulation management during IABP support.

## 2. Materials and methods

### 2.1. Study design, population, and ethical approval

This single-center retrospective cohort study systematically screened and analyzed 128 patients with CS who received IABP support at our institution between January 2023 and December 2024. All enrolled patients met the criteria for Society for Cardiovascular Angiography & Interventions (SCAI) CS stage C (classic shock) or stage D (deteriorating shock). According to the heparin concentration in the IABP flush solution, patients were divided into 2 groups: the standard-dose group received a flush solution containing 25 U/mL heparin (12,500 U heparin diluted in 500 mL normal saline), whereas the half-dose group received a flush solution containing 12.5 U/mL heparin (6250 U heparin diluted in 500 mL normal saline). Comparisons of baseline demographic characteristics, clinical characteristics, and disease severity indicators showed no statistically significant differences between the 2 groups in terms of age, sex, body mass index, or distribution of comorbidities (all *P* > .05). The patient selection process and grouping procedures are presented in Figure 1.

**Figure 1.**
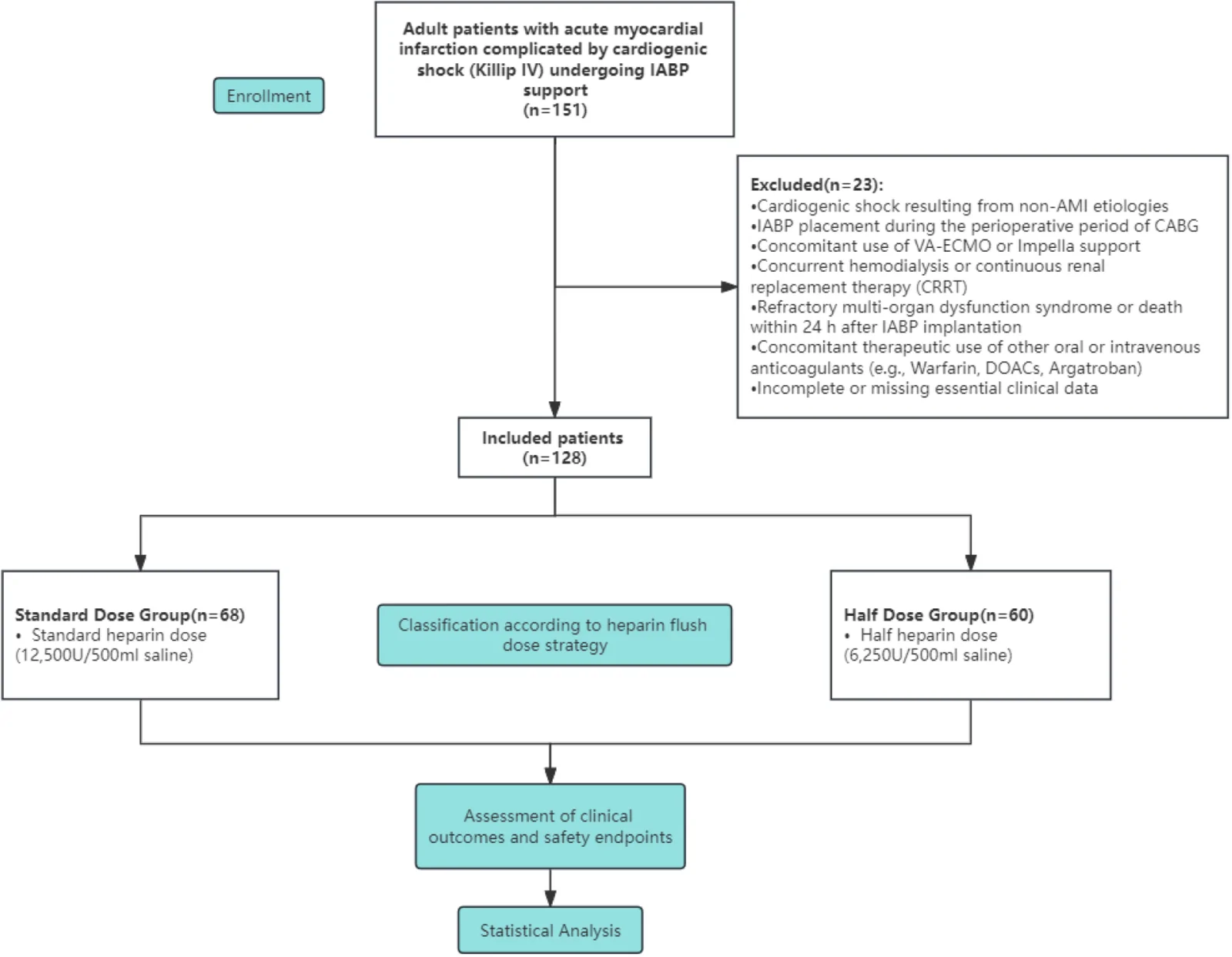
Study flowchart of patient enrollment and grouping. AMI = acute myocardial infarction, CABG = coronary artery bypass grafting, DOAC = direct oral anticoagulant, IABP = intra-aortic balloon pump, VA-ECMO = venoarterial extracorporeal membrane oxygenation.

This study was conducted in accordance with the principles of the Declaration of Helsinki and was approved by the Ethics Committee of Zhengzhou People’s Hospital (approval number: 2025-ZYLW-028). Given the retrospective nature of the study and the use of routinely collected clinical data without any experimental interventions beyond standard clinical care, the requirement for informed consent was waived by the ethics committee. All clinical data were strictly anonymized before extraction and analysis to ensure the protection of patient privacy.

### 2.2. Inclusion and exclusion criteria

To ensure the integrity and reliability of the research data, this study established multi-dimensional inclusion and exclusion criteria.

Inclusion criteria include the following: adult patients aged ≥18 years; patients who met the diagnostic criteria for acute myocardial infarction complicated by CS and had a Killip class IV status at admission were included; IABP support duration ≥24 hours; continuous catheter flushing with heparinized normal saline during IABP support, with a clear heparin dosage regimen (standard dose or half dose); and patients with extremely severe hemodynamic instability or specific end-stage organ dysfunction were excluded. These conditions were defined as follows: SCAI shock stage E, requirement for concomitant extracorporeal membrane oxygenation support, refractory shock requiring ≥3 high-dose vasoactive agents, or baseline Child–Pugh class C liver failure and primary severe coagulation disorders.

Exclusion criteria include the following: those not meeting the inclusion criteria or with incomplete clinical data; patients who received IABP placement during the perioperative period of coronary artery bypass grafting; patients who received hemodialysis or continuous hemofiltration during IABP use; death within 24 hours after surgery; those with severe hemodynamic instability, organ dysfunction, or other conditions that may affect the assessment; patients receiving other systemic anticoagulant therapies.

### 2.3. Intervention methods

All enrolled patients received standardized IABP support and peri-support management according to institutional protocols. IABP insertion was performed through the femoral artery using the Seldinger technique, and catheter positioning was confirmed under imaging guidance to ensure that the balloon tip was located below the origin of the left subclavian artery and above the renal arteries. The IABP operating mode and assist ratio were adjusted dynamically according to the patient’s hemodynamic status. During IABP support, all patients received continuous catheter flushing with heparinized saline. In addition, all patients received systemic anticoagulation according to the standardized anticoagulation protocol of our department throughout the IABP support period. For patients without contraindications to anticoagulation, continuous intravenous unfractionated heparin infusion was administered, with dose adjustments based on activated partial thromboplastin time (APTT) and activated clotting time (ACT) monitoring to maintain the target anticoagulation range. For patients with active bleeding or other contraindications to anticoagulation, systemic anticoagulation was temporarily withheld or adjusted according to clinical conditions.

Throughout the study period, both groups followed the same systemic anticoagulation strategy, including anticoagulation management during IABP support and before and after IABP removal. The only difference between the 2 groups was the heparin concentration in the IABP flush solution. Patients in the standard-dose group received a flush solution containing 25 U/mL heparin (12,500 U heparin diluted in 500 mL normal saline), whereas patients in the half-dose group received a flush solution containing 12.5 U/mL heparin (6250 U heparin diluted in 500 mL normal saline). The flush solution was continuously administered using a pressurized infusion device with the pressure maintained at approximately 300 mm Hg. The baseline flushing rate was set at 2 to 3 mL/h, and standardized rapid flushing was performed once every hour according to the clinical protocol, with a single flush volume of approximately 0.25 mL (approximately 5 drops). This minimal and intermittent flushing regimen was designed to ensure accurate catheter tip pressure monitoring and maintain catheter patency while minimizing additional heparin exposure associated with manual flushing. Given the extremely small volume of intermittent flushing, differences in heparin concentration (25 U/mL vs 12.5 U/mL) were used to modulate the local anticoagulant environment at the catheter tip without affecting the baseline systemic anticoagulation status. The flushing regimen was based on routine ICU clinical practice, and no additional interventions regarding the flushing rate or method were implemented during the study period. Hemodynamic parameters, relevant laboratory indicators, and adverse events were continuously monitored during IABP support.

### 2.4. Outcome indicators

The primary outcomes focused on anticoagulation-related safety outcomes during IABP support, including the incidence of clinically significant bleeding events, defined according to the Bleeding Academic Research Consortium (BARC) criteria as BARC type 2, 3, or 5 bleeding, including bleeding requiring medical intervention or blood transfusion, bleeding requiring surgical management or hemodynamic support, intracranial hemorrhage, and fatal bleeding; and the incidence of thrombocytopenia, defined as any platelet count <100 × 10^9^/L during IABP support.

The dynamic changes in platelet count were analyzed descriptively as a continuous variable to reflect temporal trends in platelet levels during IABP support. Platelet data were collected at prespecified time points from T1 to T5: T1 was defined as day 1 after IABP insertion (day 1), T2 as day 3 (day 3), T3 as day 5 (day 5), T4 as day 7 (day 7), and T5 as day 9 (day 9). Platelet counts at each time point were analyzed using cross-sectional comparisons between groups, and repeated-measures analyses or mixed-effects models were not applied.

Secondary outcomes included laboratory anticoagulation parameters and clinical outcomes. Laboratory anticoagulation parameters mainly included APTT and ACT. During the first 24 hours after device implantation, APTT and ACT were measured every 4 to 6 hours; after stabilization, routine monitoring was performed every morning. Dynamic anticoagulation data on days 1, 3, 5, 7, and 9 during IABP support were extracted to evaluate temporal trends. Clinical outcomes included transfusion events, in-hospital mortality, early IABP removal, and catheter-related occlusion events.

Transfusion events were defined as the administration of any blood products during hospitalization and were further categorized according to the indication as bleeding-related transfusion or non-bleeding-related transfusion. Patients who received blood products due to bleeding events meeting the BARC criteria were classified as having bleeding-related transfusions. Patients without active bleeding, defined by the BARC criteria, who received blood products for anemia, perioperative supportive care, or other non-active bleeding-related indications were classified as having non-bleeding-related transfusions.

### 2.5. Data analysis

All statistical analyses were performed using SPSS software version 27.0 (IBM Corp.). Continuous variables were initially assessed for normality. Normally distributed data were presented as mean ± standard deviation, and comparisons between groups were performed using the independent-samples *t* test. Non-normally distributed data were presented as median (Q1, Q3), and comparisons between groups were performed using the Mann–Whitney *U* test. Categorical variables were expressed as number (percentage), and comparisons between groups were performed using the χ^2^ test or Fisher’s exact test.

The between-group differences in the primary and secondary outcomes were assessed using 2 × 2 contingency tables to calculate the risk ratio (RR), risk difference, odds ratio (OR), and corresponding 95% confidence interval (CI). Both RR and OR were calculated based on 2 × 2 contingency table analyses. All clinical outcomes were independently adjudicated by 2 clinicians who were blinded to group allocation. For longitudinal analysis of platelet counts, dynamic changes in platelet levels during IABP support were evaluated at prespecified time points (T1–T5). Missing platelet measurements at later time points (T4–T5) were handled using the last observation carried forward method.

To evaluate the independent association between the half-dose heparin flush strategy and clinically relevant bleeding or thrombocytopenia, multivariable logistic regression models were constructed. Given the limited number of bleeding events in this study, a prespecified parsimonious model was applied to minimize the risk of model overfitting. Only clinically relevant confounding factors with established associations with bleeding risk were included for adjustment, including dual antiplatelet therapy and renal dysfunction (defined as serum creatinine > 133 μmol/L). Regression results were presented as adjusted OR with 95% CI. All statistical tests were two-sided, and a *P* value < .05 was considered statistically significant.

## 3. Results

### 3.1. Participant characteristics and flowchart

A total of 128 patients with CS who received IABP support in the ICU were included in this study. Among them, 68 patients received standard-dose heparin flush solution and were assigned to the standard-dose group, while 60 patients received half-dose heparin flush solution and were assigned to the half-dose group. The patient selection process and final inclusion status are presented in Figure 1.

The 2 groups showed good comparability in baseline demographic and clinical characteristics. No significant differences were observed between the standard-dose and half-dose groups in terms of age, sex, body mass index, or other baseline characteristics (all *P* > .05). In addition, the mean durations of IABP support were 14.4 days and 13.7 days in the standard-dose and half-dose groups, respectively, with no significant difference between groups (*P* = .054), indicating comparable durations of exposure to the flush solution. Detailed baseline characteristics are presented in Table 1.

**Table 1:** Comparison of patient baseline characteristics (n = 128, x ± s, n [%]).

Variable	Standard-dose group (n = 68)	Half-dose group (n = 60)	χ^2^/*t*/*U* value	*P* value
Demographics				
Age (yr)	60.92 ± 15.83	65.16 ± 15.51	−1.5251	.130
Sex, n (%)			0.0033	.957
Male	45 (66.18)	40 (66.67)		
Female	23 (33.82)	20 (33.33)		
BMI (kg/m^2^)	24.52 ± 6.14	25.03 ± 6.18	−0.4721	.637
Killip class IV, n (%)	68 (100)	60 (100)	0.0003	1.000
SOFA score	7.6 ± 1.2	7.4 ± 1.1	0.778	.438
SCAI stage, n (%)			0.005	.942
Stage C	48 (70.6)	42 (70.0)		
Stage D	20 (29.4)	18 (30.0)		
Location of myocardial infarction, n (%)			0.0323	.998
Anterior myocardial infarction	23 (33.8)	22 (36.7)		
Inferior myocardial infarction	27 (39.7)	26 (43.3)		
Right ventricular myocardial infarction	18 (26.5)	12 (20.0)		
Comorbidities, n (%)				
Hypertension	40 (58.81)	32 (53.31)	0.3953	.530
Diabetes mellitus	18 (26.52)	15 (25.17)	0.0353	.851
Coronary artery disease	51 (75.00)	46 (76.67)	0.0483	.826
Chronic kidney disease	12 (17.65)	10 (16.70)	0.0233	.883
Antiplatelet therapy			1.0713	.585
No, n (%)	25 (36.8)	27 (45.0)		
Single, n (%)	28 (41.2)	23 (38.3)		
DAPT, n (%)	15 (22.1)	10 (16.7)		
IABP (d)	14.4 ± 2.1	13.7 ± 2.2	1.949	.054
Laboratory parameters				
BNP (pg/mL)	1883 (1332–2473)	1786 (1248–2286)	22,382	.346
AST (U/L)	112.0 (59.48–156.5)	107.3 (61.92–142.1)	20,782	.860
ALT (U/L)	104.7 (51.61–161.6)	115.2 (57.91–162.3)	19,522	.676
Scr (μmol/L)	136.6 (83.38–198.8)	154.4 (106.4–190.3)	18,732	.427
D-dimer (μg/mL)	1.870 (1.368–2.623)	2.110 (1.380–2.703)	19,412	.638
APTT (s)	29.99 ± 5.80	29.23 ± 5.85	0.7201	.473
PT (s)	12.45 ± 2.07	12.98 ± 2.01	−1.4651	.145
FIB (g/L)	3.68 ± 0.83	3.53 ± 0.95	0.9621	.338
PLT (×10^9^/L)	136.3 (99.58–160.7)	130.3 (102.9–156.6)	21,762	.581
Peak troponin (ng/ml)	150.3 ± 26.1	152.7 ± 25.4	−0.517	.606
Blood gas analysis				
pH	7.36 ± 0.09	7.35 ± 0.10	0.1271	.899
Lac (mmol/L)	3.785 (2.745–4.918)	3.790 (2.293–4.813)	21,472	.611

Continuous variables are presented as mean ± standard deviation or median (interquartile range), and categorical variables are expressed as number (percentage). Group comparisons were performed using the Student *t* test or Mann–Whitney *U* test for continuous variables and the χ^2^ test or Fisher’s exact test for categorical variables.

ALT = alanine aminotransferase, APTT = activated partial thromboplastin time, AST = aspartate aminotransferase, BMI = body mass index, BNP = B-type natriuretic peptide, DAPT = dual antiplatelet therapy, FIB = fibrinogen, IABP = intra-aortic balloon pump, Lac = lactate, PLT = platelet count, PT = prothrombin time, SCAI = Society for Cardiovascular Angiography & Interventions, Scr = serum creatinine, SOFA = Sequential Organ Failure Assessment.

### 3.2. Main clinical outcomes (primary outcomes)

#### 3.2.1. Clinical bleeding events

In the primary clinical outcome, the incidence of clinically significant bleeding events in the half-dose heparin flushing group was significantly lower than that in the standard-dose group. In the standard-dose group, a total of 13 patients experienced BARC 2, 3, or 5 type bleeding events, while only 2 patients in the half-dose group had clinically relevant bleeding. In the unadjusted analysis, the incidence of clinically relevant bleeding in the half-dose group was significantly lower than that in the standard-dose group (OR = 0.15, 95% CI = 0.03–0.68, *P* = .004; Table 2). After including dual antiplatelet therapy and renal insufficiency in the multivariate logistic regression analysis, the half-dose flushing still remained independently associated with a reduced bleeding risk (adjusted OR = 0.22, 95% CI = 0.05–0.89, *P* = .033; Table 3). Most of the bleeding events were classified as BARC 2 type, and no fatal bleeding (BARC 5 type) was observed in either group (Table 4). The relevant results are presented in the form of a forest plot (Fig. 2).

**Table 2:** Primary safety outcomes and crude risk estimates.

Outcome	Half-dose (n = 60)	Standard-dose (n = 68)	Risk ratio (95% CI)	Risk difference, % (95% CI)	OR (95% CI)	*P* value
Clinically relevant bleeding	2 (3.0%)	13 (19.1%)	0.17 (0.04–0.74)	−15.8 (−26.2 to −5.4)	0.15 (0.03–0.68)	.004
Thrombocytopenia	11 (18.3%)	32 (47.1%)	0.39 (0.22–0.70)	−28.7 (−44.1 to −13.3)	0.25 (0.11–0.57)	<.001

RR, RD, and OR were calculated as half-dose versus standard-dose. RD represents the absolute risk difference (percentage points). ORs were derived from 2 × 2 contingency tables. The 95% CIs for RR and OR were calculated using the log-transformation method, and the 95% CI for RD was calculated using the Wald method.

CI = confidence interval, OR = odds ratio, RD = risk difference, RR = risk ratio.

**Table 3:** Multivariable logistic regression for primary outcomes.

Outcome	Predictor	Adjusted OR (95% CI)	*P* value
Clinically relevant bleeding	Half-dose flush	0.22 (0.05–0.89)	.033
	Dual antiplatelet therapy	1.82 (1.01–3.52)	.047
	Renal dysfunction	2.14 (1.05–4.63)	.038
Thrombocytopenia (<100 × 10^9^/L)	Half-dose flush	0.48 (0.25–0.94)	.031
	Dual antiplatelet therapy	1.22 (0.60–2.47)	.582
	Renal dysfunction	1.74 (0.91–3.42)	.094

Reference category for flush strategy: standard-dose heparin flush. Renal dysfunction was defined as serum creatinine >133 μmol/L. Dual antiplatelet therapy was defined as aspirin plus a P2Y12 inhibitor.

CI = confidence interval, OR = odds ratio.

**Table 4:** Clinically significant bleeding according to BARC classification.

Outcome	Standard-dose (n = 68)	Half-dose (n = 60)	*P* value
BARC 2	11	2	.030
BARC 3	2	0	.500
BARC 5	0	0	–
Total	13	2	.004

Bleeding events were classified according to the BARC criteria. Clinically significant bleeding was defined as BARC type 2, 3, or 5. BARC 3 includes type 3a, 3b, and 3c. *P* values were calculated using the χ^2^ test or Fisher’s exact test where appropriate.

BARC = Bleeding Academic Research Consortium.

**Figure 2.**
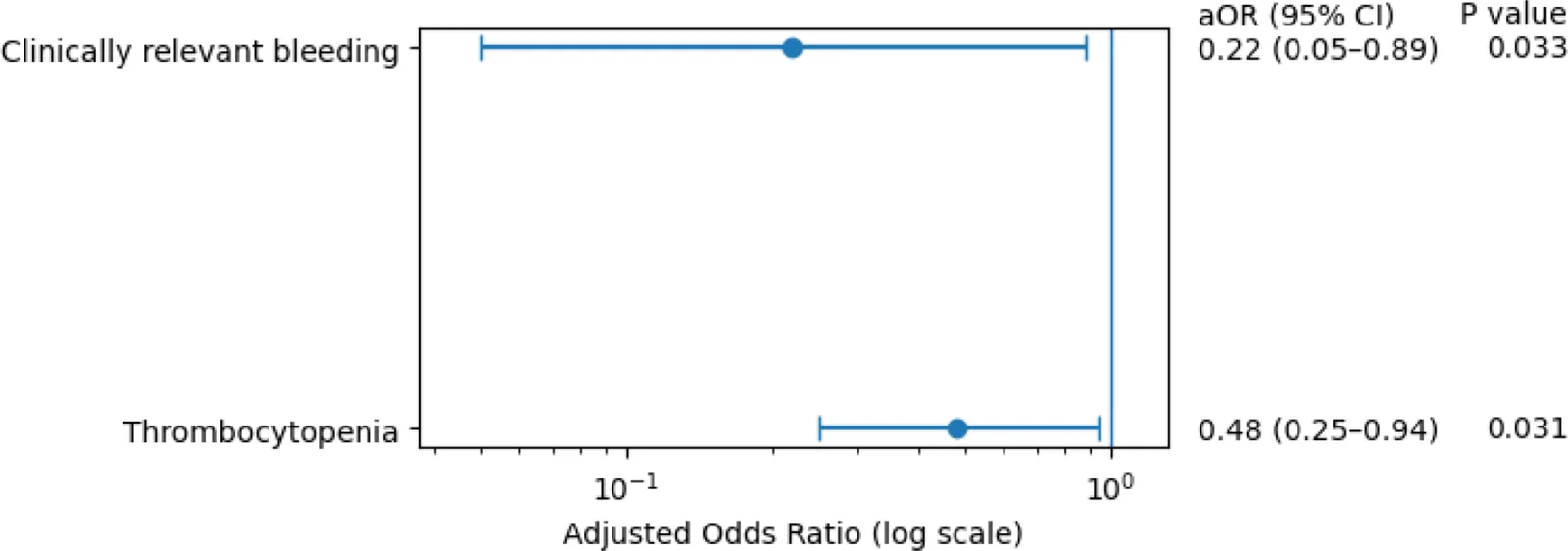
Multivariable-adjusted association between half-dose heparin flush strategy and primary safety outcomes in cardiogenic shock patients supported with IABP. aOR = adjusted odds ratio, CI = confidence interval, IABP = intra-aortic balloon pump.

#### 3.2.2. Incidence of platelet count reduction

Platelet reduction (<100 × 10^9^/L) was analyzed as one of the primary outcomes. In the standard-dose group, 32 cases (47.1%) experienced platelet reduction, while in the half-dose group, 11 cases (18.3%) did. The difference between the groups was statistically significant (*P* < .001). Unadjusted analysis showed that the half-dose heparin flushing strategy significantly reduced the risk of platelet reduction (OR = 0.25, 95% CI = 0.11–0.57). After including dual antiplatelet therapy and renal insufficiency in the multivariate logistic regression analysis, the half-dose flushing remained independently associated with a reduced risk of platelet reduction (adjusted OR = 0.48, 95% CI = 0.25–0.94, *P* = .031; Table 3, Fig. 2).

Further analysis of the dynamic changes in platelet count during IABP support between the 2 groups was conducted. At baseline (T1), the platelet count levels of the 2 groups were similar, and the difference was not statistically significant (*P* > .05). In the early stage of IABP support (T2), the platelet count of both groups decreased compared to the baseline, but the difference between the groups did not reach statistical significance (*P* > .05).

From the mid-point time of IABP support (T3), the trend of platelet count changes in the 2 groups began to show significant differentiation. The platelet count in the standard-dose group continued to decline, while the decline in the half-dose group was relatively smaller. The difference between the 2 groups reached statistical significance at T3 (*P* < .05). Subsequently, at the follow-up time points (T4 and T5), the difference in platelet count between the 2 groups persisted, and the standard-dose group maintained a lower level (all *P* < .05; Table 5, Fig. 3). Platelet count showed a clear time-dependent change characteristic during IABP support, and the difference in platelet count between the 2 groups began to emerge from T3.

**Table 5:** Platelet counts over time in the standard-dose and half-dose groups.

Time point	Standard-dose, median (P25, P75)	Half-dose, median (P25, P75)	*P* value
T1	136.3 (105.3, 160.3)	130.3 (103.7, 156.2)	.555
T2	135.1 (104.5, 159.8)	129.3 (105.0, 156.1)	.742
T3	115.2 (103.5, 128.8)	128.7 (107.5, 142.7)	.009
T4	104.9 (88.4, 118.8)	113.4 (101.4, 129.1)	.013
T5	101.7 (78.6, 112.5)	106.9 (100.9, 121.0)	.007

Data are presented as median (25th percentile, 75th percentile). *P* values were calculated using the Mann–Whitney *U* test.

**Figure 3.**
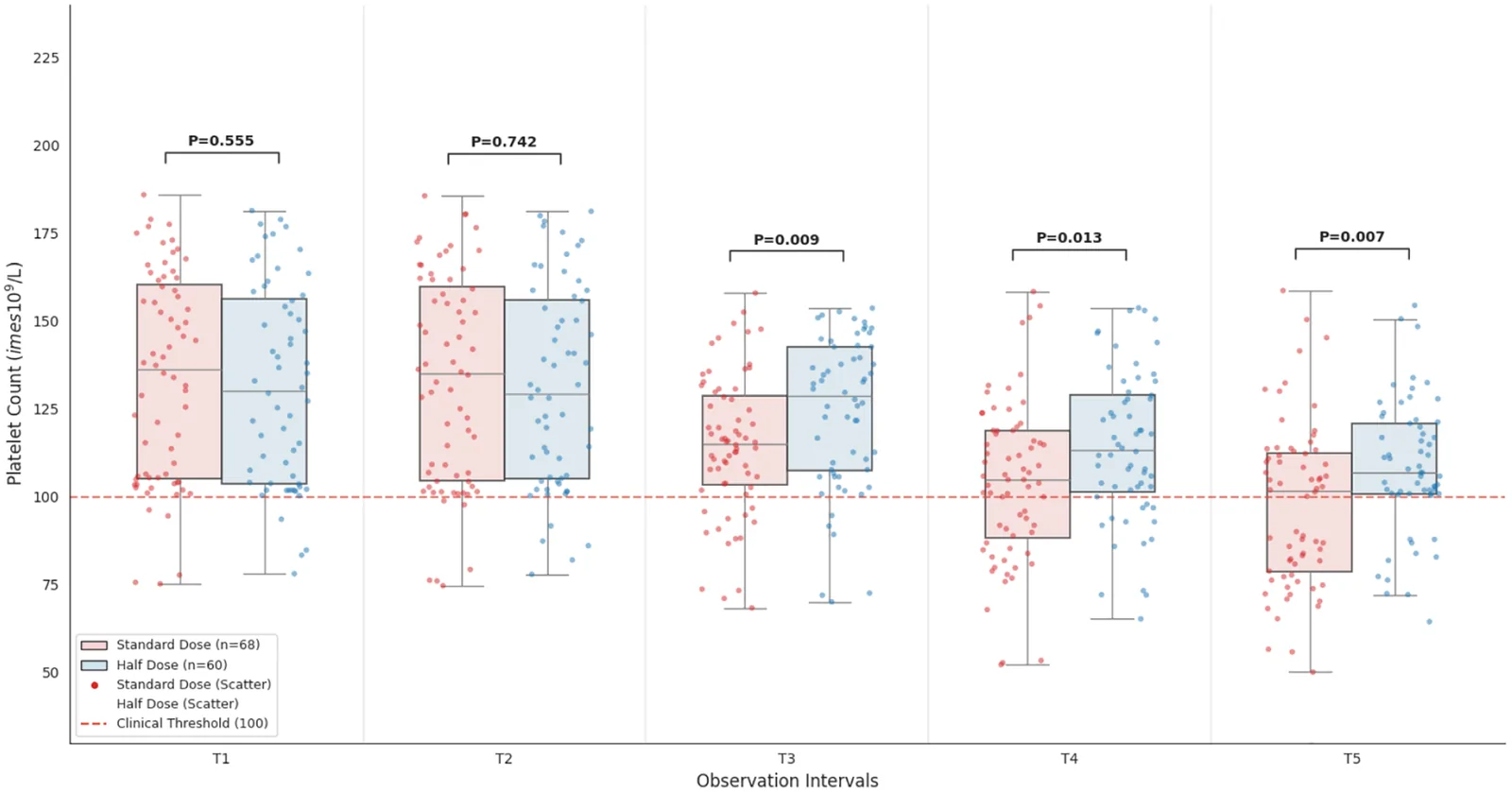
Distribution of platelet counts over time in the standard-dose and half-dose groups. T1 to T5 correspond to days 1, 3, 5, 7, and 9 after IABP insertion, respectively. IABP = intra-aortic balloon pump.

### 3.3. Secondary outcomes

The secondary outcomes were evaluated to determine whether the half-dose heparin flushing strategy, while reducing the risk of bleeding, did not affect the anticoagulation effect or the overall clinical safety.

#### 3.3.1. Laboratory anticoagulation markers

During IABP support, no statistically significant differences were observed between the 2 groups in systemic anticoagulation parameters. The comparison of APTT between the standard-dose group and the half-dose group showed no significant difference (*t* = −1.432, *P* = .154). Similarly, no significant difference was observed in ACT between the 2 groups (*t* = −0.390, *P* = .697; Table 6, Fig. 4). Throughout the study period, both groups received the same systemic anticoagulation management strategy, with the only difference being the heparin concentration in the IABP flush solution. Therefore, the laboratory anticoagulation parameters were comparable between groups, suggesting that reducing the heparin dose in the flush solution did not affect systemic anticoagulation levels or result in an apparent systemic hypocoagulable state.

**Table 6:** Comparison of systemic anticoagulation laboratory markers between groups.

Outcome	Standard-dose (n = 68)	Half-dose (n = 60)	*P* value
APTT	30.09 ± 2.92	30.90 ± 3.41	.154
ACT	80.83 ± 4.81	81.16 ± 4.75	.697

Data are presented as mean ± standard deviation. Comparisons between groups were performed using the independent-samples Student *t* test.

ACT = activated clotting time, APTT = activated partial thromboplastin time.

**Figure 4.**
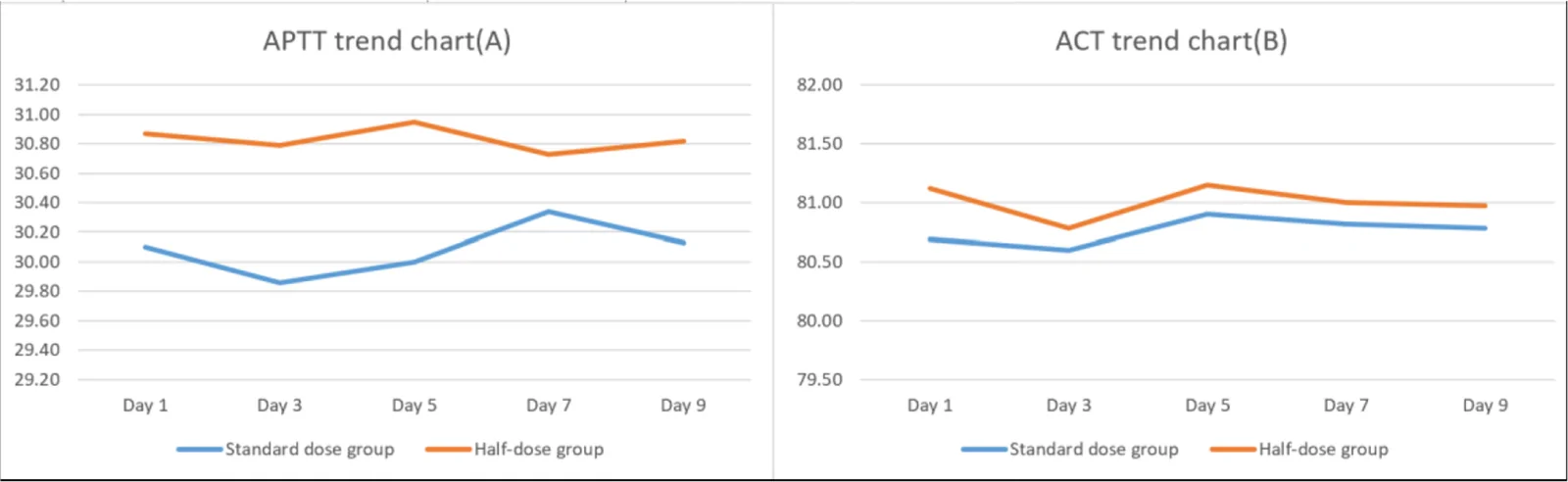
Dynamic trends of APTT and ACT during IABP support. ACT = activated clotting time, APTT = activated partial thromboplastin time, IABP = intra-aortic balloon pump.

The dynamic trend analysis demonstrated that systemic anticoagulation levels remained highly stable throughout the observation period (days 1–9) in both groups, with largely overlapping trajectories between the 2 groups (all *P* > .05; Fig. 4).

#### 3.3.2. *Secondary clinical outcomes (clinical secondary outcomes*)

In terms of secondary clinical outcomes, the transfusion requirement in the half-dose group was significantly lower than that in the standard-dose group, and the difference was statistically significant (*P* < .001). However, there was no statistically significant difference in in-hospital mortality between the 2 groups (*P* = .45). The reduction in transfusion was observed, but no difference in mortality was noted, suggesting that the bleeding benefit was not at the cost of a deteriorating prognosis. In addition, no significant difference was observed in the rate of early weaning from IABP between the 2 groups (*P* = .54; Table 7). During the study period, no catheter-related blockage events occurred in either group. Regarding the relative risk estimates of secondary clinical outcomes, compared with the standard-dose strategy, the half-dose strategy showed a clear trend of risk reduction in the transfusion outcome, while the risk estimates for mortality and early weaning from IABP were directionally neutral, and the CIs crossed 1, without showing statistically significant differences. Since no catheter blockage events occurred in both groups, the risk estimate for this outcome could not be calculated (Fig. 5). In terms of the relationship between transfusion and bleeding, there was no statistically significant difference in bleeding-related transfusions between the 2 groups; however, the incidence of non-bleeding-related transfusion was significantly higher in the standard-dose group (*P* = .008). Overall, the incidence of any reason for transfusion was significantly higher in the standard-dose group than in the half-dose group (*P* < .001; Table 8). Overall, the half-dose heparin flushing strategy reduced the burden of transfusion without observing adverse changes in short-term mortality or device safety indicators.

**Table 7:** Secondary clinical outcomes according to heparin flush dose.

Outcome	Standard-dose (n = 68)	Half-dose (n = 60)	*P* value
Transfusion	36 (52.9%)	14 (23.3%)	<.001
In-hospital mortality	5 (7.4%)	2 (3.3%)	.445
Early IABP weaning	7 (10.3%)	4 (6.7%)	.538
Catheter-related occlusion	0 (0%)	0 (0%)	–

Data are presented as number (percentage). Group comparisons were performed using the χ^2^ test or Fisher’s exact test when appropriate. Early IABP weaning was defined as successful removal of the intra-aortic balloon pump due to hemodynamic stabilization without the need for reinsertion during the same hospitalization.

IABP = intra-aortic balloon pump.

**Table 8:** Relationship between transfusion and BARC bleeding events.

Outcome	Standard-dose (n = 68)	Half-dose (n = 60)	*P* value
Bleeding-related transfusion	7	2	.173
Non-bleeding-related transfusion	29	12	.008
Any-cause transfusion	36	14	<.001

Any-cause transfusion was defined as the administration of any blood product (packed red blood cells, platelets, or plasma) during hospitalization, irrespective of indication. Bleeding-related transfusion refers to transfusion administered in the setting of BARC-defined bleeding. Non-bleeding-related transfusion includes transfusion for anemia correction, prophylactic platelet support, or other nonhemorrhagic indications. Patients may overlap between bleeding and transfusion categories.

BARC = Bleeding Academic Research Consortium.

**Figure 5.**
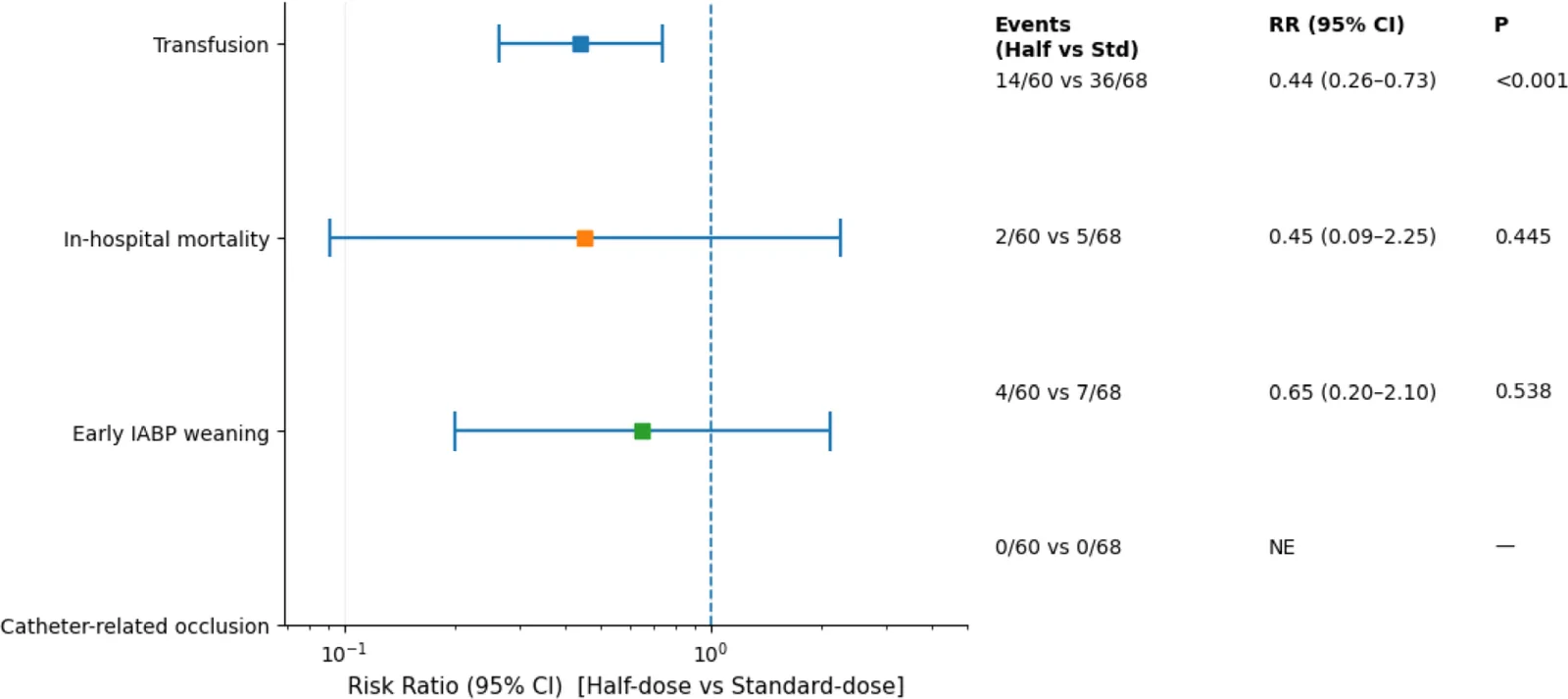
Risk ratios for secondary clinical outcomes – half-dose versus standard-dose heparin flush. CI = confidence interval, IABP = intra-aortic balloon pump, RR = risk ratio.

## 4. Discussion

### 4.1. Main findings

This study compared the clinical outcomes of different heparin flush strategies in patients with CS receiving IABP support. The main findings demonstrated that the half-dose heparin flush strategy significantly reduced the incidence of clinically relevant bleeding events and thrombocytopenia, without adversely affecting local anticoagulation efficacy, device safety, or short-term clinical outcomes.

From a risk assessment perspective, the benefits of this strategy were reflected not only in the reduction of relative risks (ORs and RRs) but also in clinically meaningful absolute risk reductions. The half-dose group showed nearly 30% and 16% absolute reductions in the risks of thrombocytopenia and clinically significant bleeding, respectively. For this extremely vulnerable high-risk CS population, these findings may have important clinical implications. Overall, reducing heparin exposure through the IABP flush solution during mechanical support may help achieve a more favorable balance between anticoagulation efficacy and bleeding safety.^[12]^

The potential pathophysiological mechanisms underlying these clinical benefits warrant further discussion. First, regarding maintenance of catheter patency, the flush solution is continuously infused at a rate of 2 to 3 mL/h under pressure from a pressurized infusion system (approximately 300 mm Hg). Its primary mechanism is the generation of a high-pressure fluid microenvironment at the catheter tip, thereby mechanically preventing blood backflow and fibrin deposition. The present findings suggest that the local anticoagulant concentration provided by half-dose heparin (12.5 U/mL) may be sufficient to reach the threshold required for thrombus inhibition and maintain the physicochemical stability of this microenvironment.

Second, with respect to bleeding risk reduction, patients with CS usually require systemic anticoagulation combined with multiple antithrombotic therapies, resulting in a coagulation system already close to a vulnerable equilibrium. Although the amount of heparin delivered through the standard flush solution appears relatively small, continuous infusion over 24 hours may lead to a clinically relevant cumulative systemic anticoagulant exposure through a potential “spillover effect.” The half-dose strategy effectively reduces this additional heparin exposure while maintaining stable routine APTT monitoring values, thereby avoiding further disruption of systemic hemostatic balance.

Finally, regarding attenuation of platelet depletion, in addition to mechanical platelet damage caused by IABP balloon inflation and deflation, unfractionated heparin itself may directly interact with platelets and induce mild nonimmune platelet activation and aggregation, a phenomenon referred to as type I heparin-induced thrombocytopenia. Reducing the heparin concentration in the flush solution may therefore mitigate this heparin-mediated pro-aggregatory effect and prevent its potential additive interaction with mechanical platelet injury. This mechanism may partly explain the more gradual decline in platelet counts observed in patients receiving the half-dose heparin flush strategy.

### 4.2. Clinical severe bleeding: risk scale and literature comparison

Bleeding is one of the most clinically impactful safety endpoints during IABP support. This study showed that the half-dose heparin flushing strategy was associated with a significant reduction in the risk of BARC 2, 3, or 5 bleeding. The RR and OR were in the same direction, and the CI did not cross the null line, resulting in internal consistency.

Previous studies on the bleeding risk during IABP support have mostly focused on systemic anticoagulation intensity or antiplatelet treatment regimens, and less on the continuous, low-dose exposure of heparin through the flushing route. This study suggests that this long-considered “background measure” of flushing anticoagulation may have independent and cumulative safety effects on bleeding risk.^[13,14]^ During IABP support, patients typically have multiple bleeding risk factors, including mechanical device-related platelet activation and consumption, dual antiplatelet therapy after acute myocardial infarction, frequent invasive procedures, and coagulation disorders caused by organ dysfunction. In this highly vulnerable context, even nontreatment-dose heparin administered through the continuous flushing route may amplify the overall bleeding risk through a time-cumulative effect. The half-dose heparin flushing strategy reduces this avoidable source of anticoagulation exposure, providing a more targeted intervention to reduce bleeding events.

The BARC classification results are shown (Table 4). Bleeding events were mainly BARC 2 type, and no fatal bleeding occurred. Although there was no statistically significant difference in BARC 2 bleeding between the 2 groups, the number of events in the half-dose group was lower than that in the standard-dose group. Considering the limited sample size and event number in the study, this difference may be affected by insufficient statistical power. However, from the perspective of risk structure, the half-dose strategy has a consistent directionality in reducing the clinical-related bleeding burden. This finding supports the necessity of dose optimization for “background anticoagulation measures” during IABP support.^[15]^

### 4.3. Thrombocytopenia: incidence and time dependency

Thrombocytopenia represents a critical concern in patients with CS, who already exhibit a high degree of clinical severity and an increased risk of multiple organ dysfunction.^[16]^ In this vulnerable population, further reductions in platelet counts may complicate antithrombotic management and increase susceptibility to bleeding. Therefore, exploring clinical interventions that can reduce platelet consumption is of substantial practical importance.

Thrombocytopenia (defined as platelet count < 100 × 10^9^/L) was another primary outcome of this study. In this study, the incidence of thrombocytopenia in the half-dose heparin flushing group was significantly lower than that in the standard-dose group (Table 2), suggesting that heparin exposure through the flushing route may be involved in the process of platelet depletion. The traditional view mostly attributes thrombocytopenia to mechanical circulatory support-related shear stress and platelet activation,^[17,18]^ while the results of this study suggest that the cumulative effect of continuous, low-dose anticoagulation exposure cannot be ignored.

It should be noted that the dynamic analysis of platelets is presented in the form of median (Q1, Q3), reflecting the distribution changes of the population, rather than all patients showing a progressive decline. Some patients maintained stable platelet counts or even experienced fluctuations and rebounds (Fig. 3), suggesting individual heterogeneity in platelet changes.

From the perspective of time trends, there was no significant difference in platelet levels between the 2 groups in the early stage of IABP implantation (T1–T2), and the changes in platelet counts were relatively stable. However, in the middle and later stages of implantation (T3–T5), they began to gradually differentiate. The standard-dose group showed an overall downward shift in platelet distribution and an expansion of the low-value range, while the half-dose group showed a relatively smaller decline. This phenomenon is more in line with the “time-dependent and cumulative effect” model rather than an immediate response.^[19]^

A more reasonable explanation is that the platelet decline shows a progressive threshold-driven characteristic. When continuous anticoagulation exposure and mechanical platelet consumption are superimposed, it is more likely to exceed the clinical threshold of <100 × 10^9^/L. The half-dose strategy may reduce the total anticoagulation exposure, possibly delaying or avoiding platelet depletion reaching this threshold, thereby reducing the incidence of platelet reduction.

### 4.4. Separation phenomenon of anticoagulation total load and laboratory indicators

It is noteworthy that the reduction in bleeding risk and the incidence of platelet reduction did not accompany significant changes in APTT or ACT. This “separation phenomenon” suggests that in the context of mechanical circulatory support, traditional coagulation time indicators may not fully reflect the comprehensive impact of low-dose, continuous anticoagulation exposure on the hemostasis system.^[20]^

The heparin in the flushing fluid enters the body through continuous pressurized infusion, forming a low-intensity but time-cumulative anticoagulation load. When this load is superimposed with mechanical shear stress, inflammatory response, and antiplatelet therapy, it may disrupt the microbalance of the hemostasis system, and this change may not be reflected in the detection of systemic coagulation time.

Therefore, this study supports an important viewpoint: during IABP support, anticoagulation management should not only focus on systemic anticoagulation intensity but also on the optimization of total anticoagulation exposure. The half-dose flushing strategy improves safety without changing conventional anticoagulation indicators, strengthening the rationality of the concept of “reducing covert anticoagulation exposure.”

### 4.5. Secondary outcome: device safety and risk structure supplementation

The secondary outcome provides external validation of the advantages of the half-dose strategy. Both groups did not experience catheter-related blockage events, and there was no significant difference in the rate of early IABP weaning, suggesting that reducing the heparin concentration in the flushing fluid did not compromise catheter patency or device function. Patients with CS present with complex clinical conditions, and risk assessment should not rely on a single parameter alone. As emphasized in previous studies, risk models such as the age, creatinine, and ejection fraction score and the Intermountain Risk Score incorporate factors including age, renal function, and shock severity, and have important value in predicting mortality risk among these high-risk patients.^[21,22]^ Furthermore, integrated multivariable prediction approaches have been successfully applied in other high-risk cardiovascular settings to facilitate early identification of vulnerable patients.^[23]^

The demand for blood transfusion was significantly reduced, which is a direct manifestation of the safety benefit at the clinical practice level. Stratified analysis showed (Table 8) that non-bleeding-related blood transfusion was more common in the standard-dose group, suggesting that the difference in blood transfusion was not solely driven by overt BARC bleeding but may be related to continuous platelet decline, subclinical bleeding, or changes in clinical risk assessment. At the same time, the impact of flushing does not only manifests in the clear bleeding endpoint but may also affect the overall blood transfusion burden by changing the structure of blood management.^[23]^

There was no difference in in-hospital mortality, indicating that the half-dose strategy improved safety without introducing detectable short-term prognostic risks or that the statistical power of this study was insufficient to detect the difference in mortality. The overall results indicate that, while maintaining device safety, the half-dose flushing strategy achieved an optimization of the risk structure.

## 5. Research advantages and limitations

The strength of this study lies in its quantitative evaluation of the clinical safety benefits associated with reducing the heparin dose in the flush solution among high-risk patients with CS undergoing IABP support. However, several limitations should be considered when interpreting our findings. First, the inherent limitations of the single-center, retrospective cohort design restrict the ability to establish definitive causal relationships. Although standardized inclusion criteria and clinical management protocols were applied and potential confounding factors were adjusted as much as possible, selection bias and residual confounding from unmeasured variables cannot be completely excluded. In addition, to minimize substantial confounding from extremely high early mortality and severe multiple organ failure when assessing cumulative anticoagulant exposure, patients with the most critical conditions, such as those with SCAI stage E, were excluded from this study. Therefore, our findings are primarily applicable to relatively stable patients with typical CS receiving IABP support, such as those with SCAI stage C or D. For patients at the threshold of profound hemodynamic collapse, anticoagulation strategies should be considered on an individualized basis. Second, the relatively limited sample size, together with the extremely low incidence of adverse events such as catheter occlusion, resulted in insufficient statistical power to formally establish the non-inferiority of the half-dose strategy for maintaining catheter patency. Furthermore, the assessment of systemic anticoagulation status relied primarily on routine laboratory parameters (APTT and ACT), without systematic evaluation of microvascular thrombosis or specific immunological markers. Finally, the observation period was limited to hospitalization, precluding assessment of the potential effects of the different heparin flush strategies on intermediate- and long-term clinical outcomes.

Given these limitations, large-scale, prospective, multicenter randomized controlled trials are warranted to further validate the safety and clinical applicability of the half-dose heparin flush strategy. First, conventional anticoagulation management during the peri-IABP period may vary among medical centers. In the present study, both groups received a standardized systemic anticoagulation management protocol; therefore, the observed differences were more likely to reflect the effects of different heparin concentrations in the flush solution. However, because systemic anticoagulation regimens, target ACT/APTT ranges, and anticoagulation management during IABP weaning may differ across institutions, the generalizability of our findings to other centers should be interpreted in the context of local anticoagulation practices. Multicenter studies could help overcome the limitations of single-center experience and determine whether the half-dose flush strategy is applicable across broader real-world settings. Second, rigorous randomization would help minimize baseline imbalances and provide stronger evidence regarding the causal relationship between anticoagulation de-escalation and bleeding outcomes. Finally, adequately powered prospective clinical trials with a predefined non-inferiority margin are needed to determine whether reducing local heparin exposure can maintain device patency without increasing the risk of device-related thrombosis. High-quality evidence from such randomized controlled trials could provide an important basis for the future standardization and refinement of anticoagulation management strategies in the field of mechanical circulatory support.

## 6. Conclusion

Among patients with CS receiving IABP support, our findings suggest that, compared with the standard-dose strategy, the half-dose heparin flush strategy was associated with lower incidences of clinically significant bleeding, thrombocytopenia, and transfusion requirements. By reducing heparin exposure through the flush solution, the half-dose strategy did not result in significant changes in systemic anticoagulation levels. No catheter occlusion events were observed in either group; however, given the limited sample size and the low incidence of catheter occlusion, the non-inferiority of the half-dose strategy regarding device safety cannot be established based on the current findings.

These results suggest that optimization of the heparin concentration in the IABP flush solution may represent a potential strategy for reducing anticoagulation-related complications during IABP support. Nevertheless, as this was a single-center retrospective study, further large-scale, multicenter prospective studies are required to validate these findings and to determine the optimal anticoagulation management strategy across different durations of IABP support and patient subgroups.

## Acknowledgments

The authors sincerely thank all physicians, nurses, and staff of the intensive care unit at Zhengzhou People’s Hospital, Zhengzhou, China, for their dedication to patient care and their assistance in data collection. The authors are also grateful to the clinical research team for their support in data management and statistical consultation.

## Author contributions

**Conceptualization:** Yunkai Li, Weixia Yang, Dalong Zhang.

**Data curation:** Yunkai Li, Yiwei Yu, Bingbing Fan, Xingguo Niu.

**Formal analysis:** Yunkai Li, Yiwei Yu.

**Investigation:** Yiwei Yu, Bingbing Fan, Huijun Dong, Yizhe Wang, Xingguo Niu.

**Methodology:** Yunkai Li, Weixia Yang.

**Project administration:** Weixia Yang, Dalong Zhang.

**Resources:** Hanxiao Nie, Zhengtao Hao, Zhenzhen Wang.

**Software:** Yiwei Yu.

**Validation:** Weixia Yang, Huijun Dong.

**Visualization:** Yunkai Li, Yizhe Wang.

**Writing – original draft:** Yunkai Li, Weixia Yang.

**Writing – review & editing:** Weixia Yang, Dalong Zhang.
